# Indoor Mapping of Magnetic Fields Using UAV Equipped with Fluxgate Magnetometer

**DOI:** 10.3390/s21124191

**Published:** 2021-06-18

**Authors:** Pavol Lipovský, Katarína Draganová, Jozef Novotňák, Zoltán Szőke, Martin Fiľko

**Affiliations:** Faculty of Aeronautics, Technical University of Košice, Rampová 7, 041 21 Košice, Slovakia; pavol.lipovsky@tuke.sk (P.L.); jozef.novotnak@tuke.sk (J.N.); zoltan.szoke@tuke.sk (Z.S.); martin.filko@tuke.sk (M.F.)

**Keywords:** magnetic field, magnetic sensor, unmanned aerial vehicle, mapping

## Abstract

Unmanned aerial vehicles (UAVs) are used nowadays in a wide range of applications, including monitoring, mapping, or surveying tasks, involving magnetic field mapping, mainly for geological and geophysical purposes. However, thanks to the integration of ultrasound-aided navigation used for indoor UAV flight planning and development in sensorics, the acquired magnetic field images can be further used, for example, to enhance indoor UAV navigation based on the physical quantities of the image or for the identification of risk areas in manufacturing or industrial halls, where workers can be exposed to high values of electromagnetic fields. The knowledge of the spatial distribution of magnetic fields can also provide valuable information from the perspective of the technical cleanliness. This paper presents results achieved with the original fluxgate magnetometer developed and specially modified for integration on the UAV. Since the magnetometer had a wider frequency range of measurement, up to 250 Hz, the DC (Direct Current) magnetic field and low frequency industrial components could be evaluated. From the obtained data, 3D magnetic field images using spline interpolation algorithms written in the Python programming language were created. The visualization of the measured magnetic field in the 3D plots offer an innovative view of the spatial distribution of the magnetic field in the area of interest.

## 1. Introduction

Nowadays, unmanned aerial vehicles (UAVs) are not only used in the military, but also in a wide range of civil applications. If we consider their use in aerial work, they can be used, for example, in monitoring, mapping, or surveying purposes in many types of industrial, search and rescue, security, or safety purposes, even in challenging or dangerous conditions [1,2,3,4]. The current study also deals with the possibilities of their use in smart cities [5] and smart buildings [6].

If we focus on the UAV applications dealing with magnetic field measurement or mapping, the research is mainly focused on the cost-effective airborne magnetic surveys for industrial and geoscientific applications [7] in exteriors. These applications not only involve geological and geophysical mapping [8], which can be performed thanks to the UAVs on a regional scale [9], but can also be used to perform magnetic surveys for geothermal exploration [10], volcano monitoring [11], and related geohazard assessments [12] for the evaluation of factors affecting gully headcut locations [13]. The magnetic sensors placed on the UAV boards can be used for mineral exploration and mining operations [14] or for the detection and identification of the orphaned oil and gas wells [15]. The aeromagnetic measurements [16] can be integrated with other types of sensors, for example, with lidar [17] or photogrammetric cameras [18,19,20]. However, nowadays, geospatial analytics of magnetic fields are usually performed in the outdoor environment [21].

The indoor applications of UAVs are usually related to search and rescue applications [22] involving people or object detection in cluttered indoor environments [23] or UAV-based emergency monitoring and response systems for indoor hazard monitoring and building evacuation purposes [24]. They can be used for the location of the causes of emergency situations, for example, gas sources [25]. If we consider magnetic sensors, they are usually only used as a support for indoor navigation systems [26] because due to missing signals from satellite navigation systems, indoor navigation is one of the more challenging problems that need to be solved regarding the ongoing extensive research in the area of UAVs [27,28,29,30]. Therefore, many types of visual-based [31,32,33], visual-aided [34], acoustic [35], and ultrasonic methods [36] and the related integrated navigation, localization [37,38], positioning and landing systems [39,40,41], indoor path-planning [42], and mapping guidance algorithms [43] have been developed. There are only a few experimental systems that are based on the magnetic sensors used for navigation purposes [44,45,46,47] since the field has to be mapped with sufficient precision and sensitivity.

The idea of magnetic field mapping in the indoor environment, which is presented in this article, has its origin in the ongoing continuous research and development of magnetic sensors and their applications, which also involves the concept of a magnetometer for UAV geophysical survey [48]. In combination with the knowledge obtained during the research in the area of analysis of small UAV electromagnetic images in a very low frequency range [49] together with the performed magnetic field measurements related to safety [50,51], we proposed the original idea that small UAVs could be used for quick magnetic field mapping and evaluation, not only outdoors, but thanks to the ultrasonic-aided navigation, could be used in the indoor environment as well. As mapping nowadays is performed mostly for external environments, often in large areas, the challenges in these areas involve the integration of the measured and post-processed results onto maps. The visualization has to respect the terrain and relief of the environment [52,53], many times for archaeological purposes [54,55]. The aerial data [56] also maps for subsurface structural monitoring [57]. Sometimes, the research is focused on only the visualization of the measured magnetic field data [58,59,60], often for the purposes of electromagnetic compatibility [61], using different kinds of software solutions [62,63] and different visualization methods involving for example, visualization using flux lines [64], volumetric display [65], or even interactive visualization methodologies [66,67].

This paper presents the original results of the 3D DC and industrial AC magnetic field mapping experiment performed by a small multi-rotor custom UAV in an indoor environment. The measurements were performed using our own developed vector fluxgate magnetometer since for the creation of precise magnetic map devices with the sensitivity of 5 nT or better with sufficient bandwidth, DC up to at least the 200 Hz frequency has to be used. It is very difficult to meet these parameters with the currently existing UAV systems, especially if we consider the MEMS devices used in other published experiments. In our case, it is possible to measure and visualize the magnetic field not only in the form of 2D maps, but also 3D maps while using the small UAV. Considering the technical aspect, this paper presents 3D visualization of the magnetic fields in the form of 3D spatial graphs with isosurfaces, which offer a quickly understandable insight into the 3D distribution of the magnetic field in volume. This approach is not often used despite the fact that it is quickly understandable and readable by the technical personnel evaluating the results. The obtained and postprocessed 3D measurement results can be further used, for example, as a basis for UAV navigation using the integration of different physical quantities or for the purposes of the above-mentioned analysis of magnetic fields in large-size manufacturing or other industrial buildings that have to be inspected due to the possible risks arising from the exposure of workers to the electromagnetic fields [68,69,70,71,72,73] that can influence their health [74,75,76,77,78] and safety [79,80,81].

The outline of the paper is as follows: In Section 2. we introduce the small UAV in Section 2.1 and the positioning system used during the indoor experiments, which is described in Section 2.2. We then present the relax-type fluxgate magnetometer VEMA that was developed at the Technical University of Košice and was modified to be usable with the introduced UAV (Section 2.3). The experiment site and the data acquisition method are introduced in the Section 2.4. The next section, Section 3, first presents the results of the verification measurements of the DC magnetic field performed in a plane with the experimental version of the VEMA magnetometer and with the commercial LEMI-011 magnetometer (Section 3.1). In the following section, Section 3.2, the results of the 3D DC and industrial AC magnetic field measurements performed at the same time with the introduced UAV equipped with the VEMA magnetometer are visualized and discussed. This section also contains discussion on the usability of the acquired data. In the last section, Section 4, the conclusions based on the experiment results are summarized.

## 2. Materials and Methods

A customized UAV with ultrasonic-based flight trajectory planning was used for magnetic field mapping in the indoor environment. For the magnetic field measurements, the developed VEMA magnetometer was used. For the verification comparison of the DC magnetic fields mapping results, the measurements were also performed using the commercial LEMI fluxgate magnetic sensor. However, the higher industrial frequencies were only measured with the VEMA device since it provided the sufficient frequency range of up to 250 Hz.

### 2.1. Unmanned Aerial Vehicle

A custom small UAV of the quadcopter type with a 3D-printed frame with attachment points for magnetometer assembly was used for the measurements. The propulsion system of the UAV consisted of four RAY G3 C2830-1300 BLDC motors with RAY G2 30A regulators and 9 × 5 inch three-bladed propellers. The Pixhawk 4 Mini board was used for flight control, the board ran Ardupilot Copter 4.0.7 firmware in combination with the Marvelmind ultrasound beacons for precise indoor positioning, and autonomous flight mode was used. The flight battery was a 4500 mAh 3S LiPo, which also supplied 12 V power to the magnetometer. In this configuration, the dry weight of the quadcopter (without the payload) was 1.4 kg with a maximum thrust of 3.5 kg and 15 min of flight time. This means that the UAV can safely operate, even with the added weight of the magnetometer.

**Figure 1:**
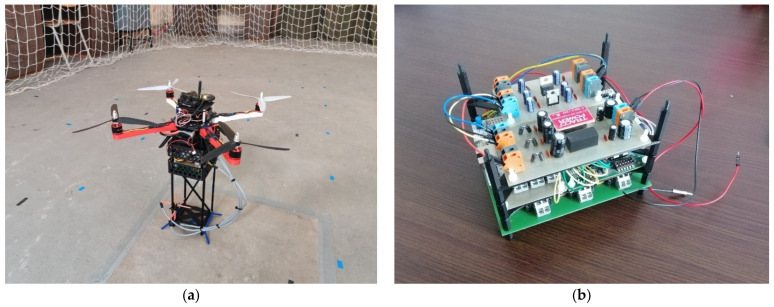
Customized UAV (Unmanned Aerial Vehicle) for magnetic field measurement: (**a**) before the measurement in the UAV Testing Hall; (**b**) details of the electronic measurement unit.

One of the tasks that had to be completed was the reduction of the influence of the interfering magnetic fields created by the motors on the measurements. Based on the knowledge and experience obtained during the research focusing on UAV detection using magnetic sensors [49], an extended landing fixture with the length of 40 cm was designed and created using the 3D printing technique. This fixture allowed the magnetic sensors to be placed on the bottom, whereas the electronic sensors were placed just under the UAV. A light-weight 3D-printed case was also created to house all of the experimental electronic components. The customized UAV for the magnetic field measurement together with the details of the experimental electronics unit can be seen in Figure 1.

### 2.2. UAV Positioning System

**Figure 2:**
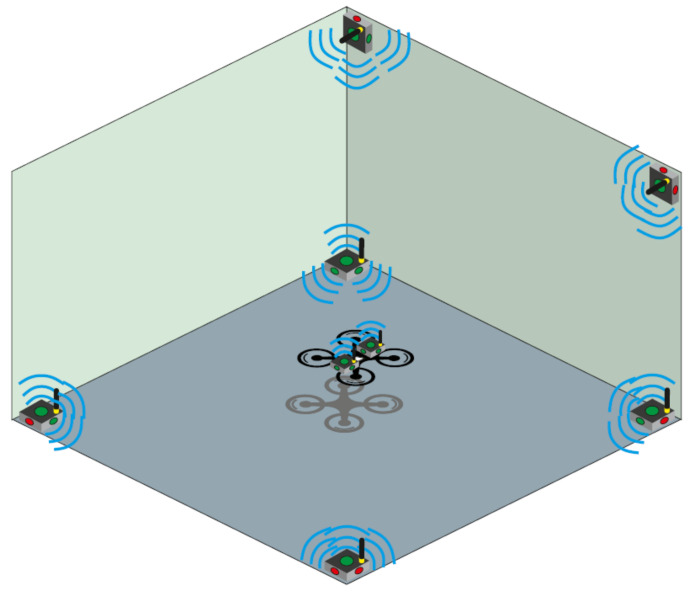
Concept of the ultrasound mobile beacon placement.

One of the crucial problems when considering UAV application in indoor environments is the missing GNSS (Global Navigation Satellite System) signal that is commonly used for flight trajectory planning in the autonomous or semi-autonomous modes in exteriors. As we assumed the use of the UAV in a known environment, we did not have to solve any challenging problems involving obstacle avoidance; therefore, the UAV was planned to fly in the autonomous mode using the position data from the Marvelmind positioning system. This system uses the ultrasound propagation time-of-flight principle using four or more stationary beacons to calculate the locations of the mobile beacons mounted on the tracked object with an accuracy of ±2 cm. The positioning of the mobile beacons used during the experimental measurements can be seen in Figure 2. The mobile beacons can also work in pairs to give information regarding the orientation of the object. There is a cross-platform compatibility of the Ardupilot and Marvelmind systems, which allows the quadcopter to use the ultrasound-based position instead of the GPS source, enabling autonomous flight. For this, one of the mobile beacons mounted on the UAV was connected through a UART interface to the Pixhawk 4 Mini autopilot.

There are two possible methods for the configuration of the Marvelmind system as a position source for the Ardupilot. Ifthe first method is applied, the geographical location coordinates of the beacon system need to be inserted in the Marvelmind software, and the correct orientation also has to be set. The mobile beacon is set to transmit the NMEA (National Marine Electronics Association) format position data to the flight controller, and it is set to accept these data as a primary GPS source. Applying this methodology, the autopilot works as if it was using the GPS for position determination.

If the second method is applied, the beacon system is set up using the default parameters, and the mobile beacon transmits the Marvelmind format data to the autopilot with its 3D spatial position and the distances to all of the stationary beacons. The calculation of these data to the geographical location is completed using the Ardupilot software. For the experiments, the second method was used as it enables the flight controller to be aware of the use of ultrasound positioning instead of the GPS.

With the correct set-up of the systems, the UAV appears on the map in the ground control software in the correct location. As it is difficult to create a precise flight plan in such small areas, the method recommended by the Marvelmind manual was used. The quadcopter was manually placed on the ground at all the waypoints, and the positions were recorded in the GCS (Ground Control Station). Afterwards, the correct flight altitude for the waypoints was set, and the start and end procedures of the mission were configured.

The UAV flew in a zig-zag pattern with 2 s stops above each measurement point. A new flight battery was used for each measured layer. The native log from the Marvelmind Dashboard was used to log the positions, and it was synchronized with the logs from the magnetometers using the system time.

### 2.3. Magnetometer

The experimental modified VEMA magnetometer was used for indoor magnetic field mapping in the performed experiments. VEMA is a relax-type fluxgate magnetometer developed at the Department of Aviation Technical Studies at the Faculty of Aeronautics of the Technical University of Košice.

**Figure 3:**
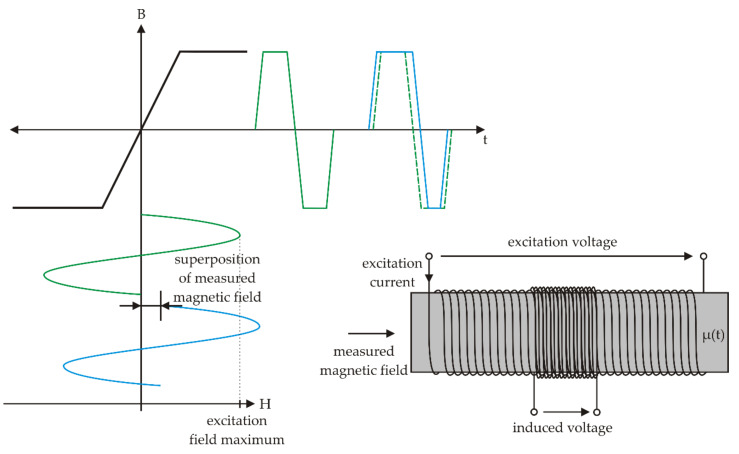
Simple fluxgate operational principle.

**Figure 4:**
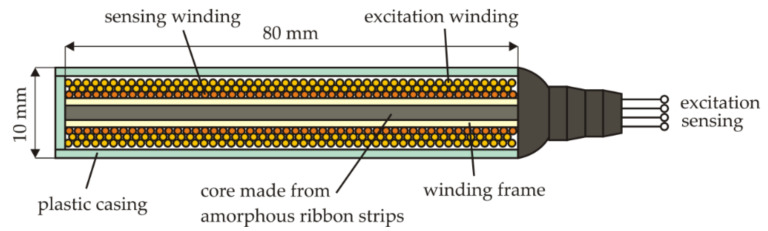
Standard VEMA sensor geometry.

The VEMA magnetometer belongs to the fluxgate magnetometers family since it also utilizes the gating of the magnetic flux in the sensor. However, compared to the general fluxgate functioning principle, it utilizes the measurements of the transient effects duration; therefore, it operates with the conversion of the magnetic field measurements to time measurements. Considering the basic general fluxgate sensor operation illustrated in Figure 3, the simple fluxgate with an open core is not often used as there is a strong signal on the excitation frequency sensed at the sensing coil output since the sensor behaves like a transformer. Therefore, two rod, ring, or racetrack cores are usually used. However, this configuration complicates the sensor fabrication process. The VEMA sensor is illustrated in Figure 4. It consists of two concentric coils, excitation and sensing, with cores made from six to ten amorphous ribbons strips, VITROVAC VAC6025 or VAC6030, made by the VACUUMSCHMELZE company. The dimensions of one strip are 80 mm × 1.5 mm or 2 mm × 25 μm or 30 μm. The excitation coil winding has twice the turns compared to the sensing coil winding. This kind of sensor geometry is easy to manufacture and handle and can be effectively used according to the operational principles of the VEMA device. 

**Figure 5:**
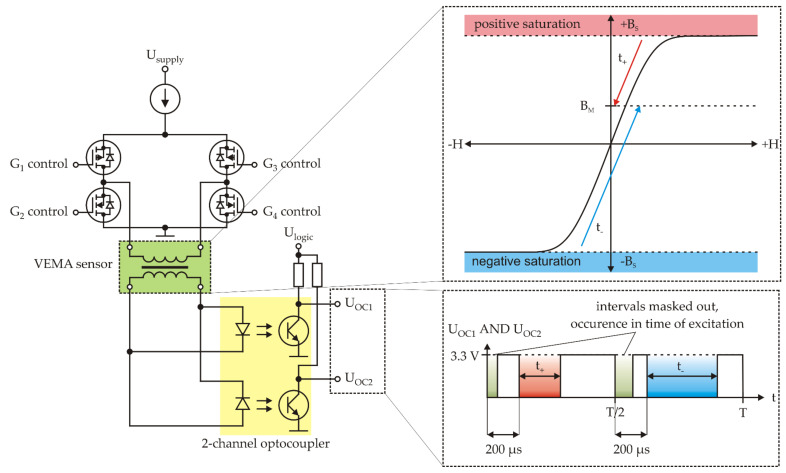
Three-state current source and evaluated signals.

The transient effects are initiated by the periodical saturation of the core by the rectangular current pulses generated by the three-state current source powering the excitation bridge, as shown in Figure 5. From this point of view, if we consider the simplified linear model of the core and the measured magnetic field *B_M_* to be smaller than the technical saturation *B_S_* of the core, for the initial value of the current we can write:

(1) $$I_{0}=\frac{l}{n\mu_{0}}\left(\frac{B_{S}}{\mu_{ef}}-B_{M}\right),$$

where *n* is the number of the sensing winding turns, *l* is the length of the sensor, and *μ*_0_ and *μ_ef_* are the vacuum permeability and effective permeability of the core material. If we consider the parametric load of the sensing winding for the simplicity created by an ideal diode with the *U_T_* threshold voltage, when the current *i_D_* flowing through the diode is larger than zero, we can write relationships:

(2) $$i_{D}=I_{0}-\frac{U_{T}}{L}t,\;u_{D}=U_{T}.$$

**Figure 6:**
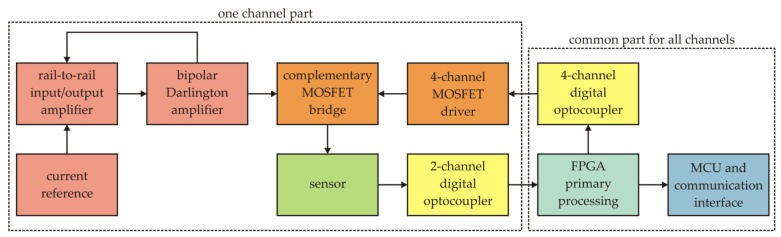
Block diagram of the measurement chain of one channel.

The gates of the bridge are controlled by the small FPGA (Field Programmable Logic Device) MAX 10 from Intel in such a manner that they create alternating pulses in both directions (polarities with respect to the coil) with a 200 μs duration and with the repeating frequency of each polarity of 500 Hz while the negative pulses are shifted of 180° with respect to the positive pulses. The response is parametrized by the optocouplers with the digital output in the 3.3 V logic, and these optocoupler signals are then merged with the AND function and further processed by the microcontroller (MCU), currently with the ARM Cortex-M7 core (Figure 6). Considering Equations (1) and (2) and the operational principle from the Figure 5, the value of the measured magnetic field is proportional to the measured difference of time intervals:

(3) $$B_{M}=k\left(t_{+}-t_{-}\right)\;+\;q.$$

where *k* and *q* are the sensitivity and offset constants, respectively, which are determined experimentally for every measurement channel during the calibration process.

**Figure 7:**
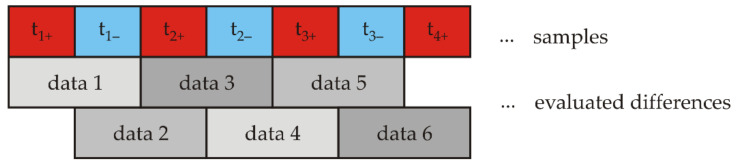
Interleaving of samples.

The sample rate of the used VEMA magnetometer is 1000 Hz since the difference of the time intervals is evaluated twice within the 500 Hz excitation signal frequency (Figure 7). The frequency bandwidth is DC—250 Hz (−3 dB).

**Figure 8:**
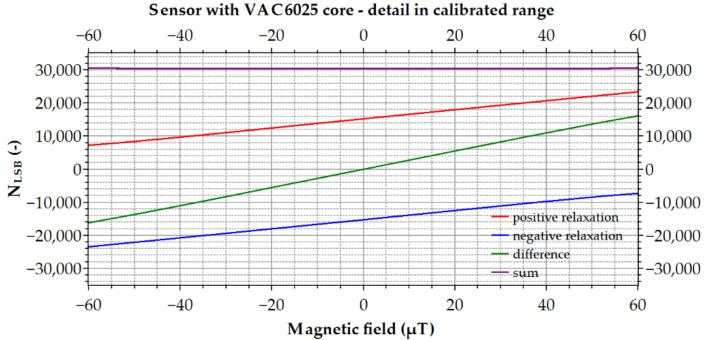
Block diagram of the measurement chain of one channel.

An example of the transfer characteristics of one channel is shown in Figure 8, where the values of positive and negative time intervals and their differences and sums are shown. The sum of the measured time intervals is used for the thermal compensation of the measurements since the threshold voltage of the diode is temperature dependent, and the increase or decrease of its value is exhibited in the value of the sum; thus, no external thermometer is necessary. It is also necessary to mention that the three VEMA magnetometer channels are sampled and evaluated simultaneously, so in the measurement point, the phase properties among the magnetic field components can also be evaluated.

There are many calibration methods that can be used for magnetometer calibration [82,83,84,85,86,87,88]. In our case, the magnetometer was calibrated using the neural network algorithm [88] and noise analysis methodology [89] developed at our department, which was also applied for the onboard satellite magnetometer calibration, described in detail in [88], with 500 calibration points of the total magnetic field of 60 μT generated with equal distribution on a virtual sphere surface. The achieved sensitivity was approximately 3.5 nT/LSB for all channels; only small differences among the channels were observed, and the internal counters in the FPGA were clocked with the 200 MHz signal; thus, the LSB was equal to 5 ns. Offsets were smaller than ±40 nT. All channels had their calibration constants implemented in the microcontroller.

The calibration process was performed with the VEMA magnetometer mounted in-place, and although the UAV construction was created mostly from non-magnetic materials, some parts still contained ferromagnetic components that could cause offsets. Precautions were also made based on the previous investigation of possible interference from the UAV propulsion system [49]; therefore, the sensors were distanced from the UAV on the fixture at the experimentally determined distance.

### 2.4. Magnetic Field Mapping and Postprocessing of the Measured Data

**Figure 9:**
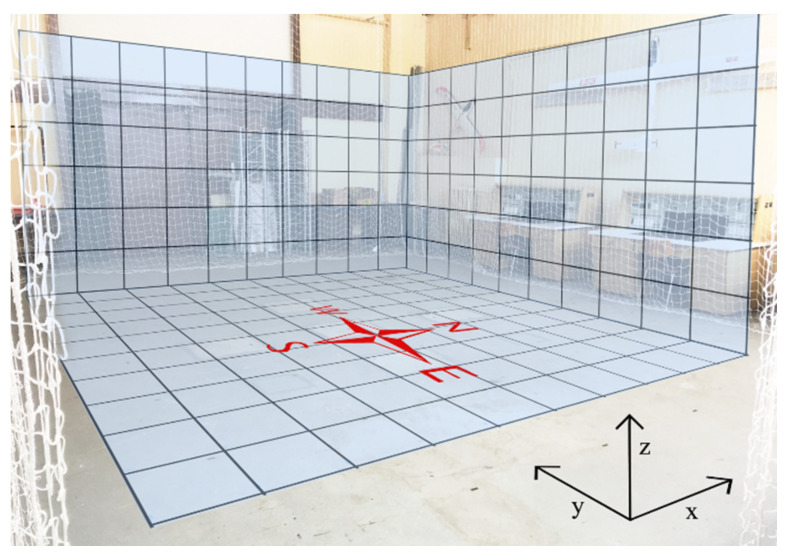
The measurement location—the Hall for UAS (Unmanned Aerial System) Testing.

For the test measurements performed with the magnetometer, the grid mapping approach was chosen. Since the tested magnetometer was placed under the quadcopter on a firm fixture, by choosing a regular orthogonal grid with the leveled position hold for two seconds, during which the measurements were performed, the possible complications arising from the uncertainties caused by the pitch, roll, and yaw angles were eliminated. Therefore, no data correction was required. The spacing between the measurement points in the grid was 0.5 m along each axis. The UAV was held in the same orientation with respect to the chosen coordinate system shown in Figure 9 for each measurement point during the whole experiment.

In case of the 2D visualization, the recorded data were postprocessed using the Matlab software. The innovative original visualization of the magnetic field in 3D space was performed using scripts in the Python programming language. For the interpolation and visualization of the measured data in 3D space, the Scipy and Plotly libraries were used. For the interpolation methodology, cubic splines using four computed interpolated values between two known measured points were applied. For the convenient 3D visualization of space with preserved depth perception, 50 surfaces with the opacity value of 0.3 were set. For the DC magnetic fields, linear scaling of the opacity was used, and for the AC fields with the 50 Hz and 150 Hz frequencies, the scaling of opacity to the maximal values was used.

The measurements were performed in the hall for UAS (Unmanned Aerial Systems) Testing at the Department of Aviation Technical Studies at the Faculty of Aeronautics. The hall together with the measurement grid and cardinal points of the magnetic compass can be seen in Figure 9.

## 3. Results and Discussion

### 3.1. Verification Test Measurement in a Plane

**Figure 10:**
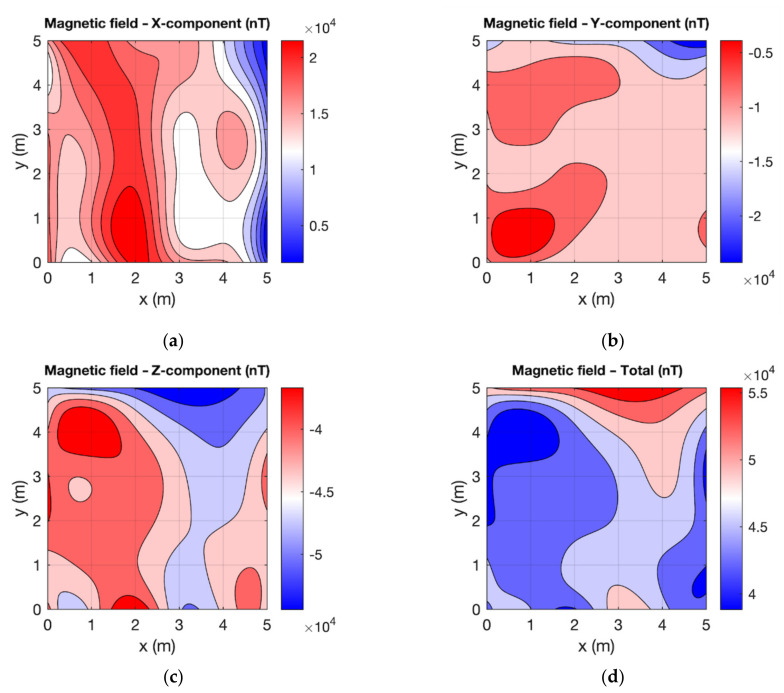
Magnetic field measured by the VEMA magnetometer: (**a**) X-component; (**b**) Y-component; (**c**) Z-component; (**d**) total.

**Figure 11:**
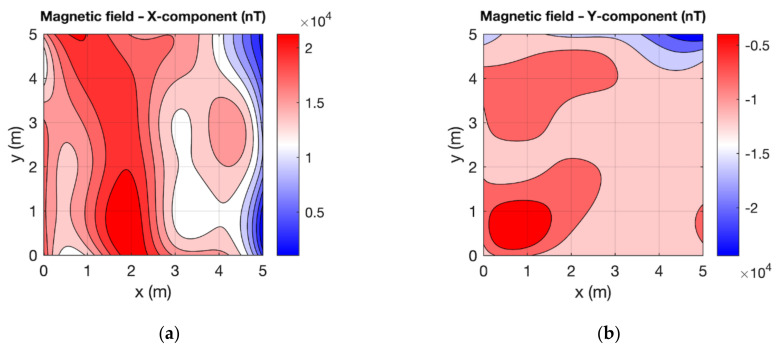

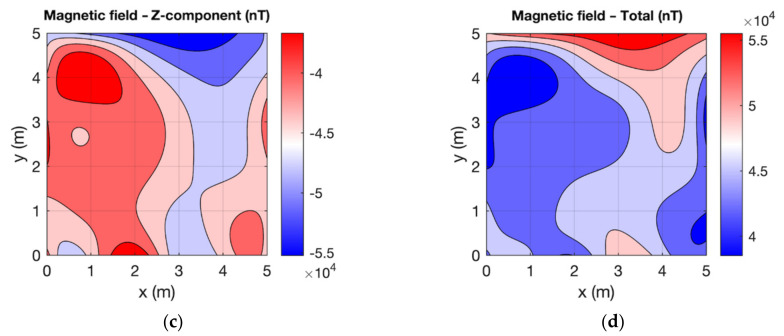
Magnetic field measured by the LEMI magnetometer: (**a**) X-component; (**b**) Y-component; (**c**) Z-component; (**d**) total.

In order to verify the correct functionality of the experimental version of the VEMA magnetometer, we performed the verification measurements using the commercial LEMI-011 magnetometer, which served as the reference measurements. The measurements were performed using the UAV in the measurement points with a grid of 6 by 6 points. The measurement spacing between the data was 1 m in both directions. The measuring height was 0.5 m above the floor. To plot the measured values, spline interpolation was used. From the graphs in Figure 10 and Figure 11, it can be seen that the results measured with two different magnetometers show a similar distribution of the magnetic field, considering the possible error in position within the range of ±2 cm.

### 3.2. Volume Measurements with the VEMA Magnetometer

**Figure 12:**
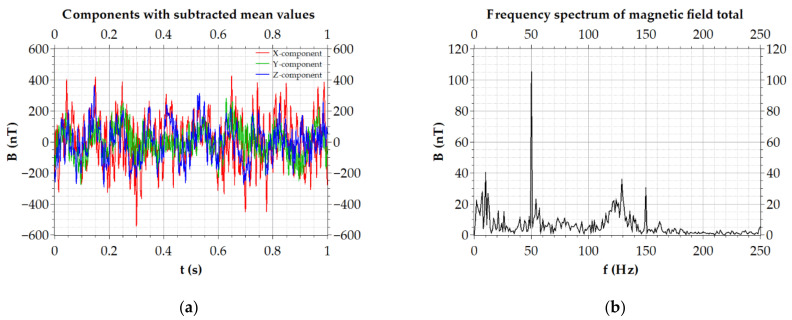
Example of 1 s signal recording: (**a**) time development of the magnetic field components; (**b**) spectrum of the corresponding magnetic field total.

After the verification measurements, the mapping of the magnetic field in volume with the dimensions of 5 m × 5 m × 2.5 m was performed. An example of the signal recording of the measurement points is shown in Figure 12. As it can be seen, although the signal is rather noisy and contains interference from the quadcopter propulsion system, through spectral analysis, the industrial AC magnetic fields can also be evaluated. 

The interpolations for the 3D visualizations were performed sequentially for each component of the measured field along each positioning coordinate system axis in the X, Y, Z order. In the first sequence, interpolation was only performed between the known points (segments) along the X axis. The second sequence filled the interpolated values of the magnetic field components along the Y axis, creating a fully interpolated plane of the values. The third sequence, along the Z axis, created interpolations among the XY planes, completing the interpolation of the volume. For verification purposes, different ordering of the axes in interpolation sequences were also tested without any noticeable differences. In our case, the cubic spline interpolation was applied. Using spline interpolation ensured that the interpolated curve had to involve the measured points. Using this method, the approximated data do not modify the measured data; therefore, the information regarding the values of the magnetic field cannot be dynamically modified. Thanks to this proposed methodology, it is possible to visualize the magnetic field in 3D space and to offer a unique technical view on the spatial distribution of the magnetic field components, which, together with the experimental design of the VEMA magnetometer and modified UAV construction, is one of the main contributions of the paper. This visualization methodology can be used for the visualization of any spatial magnetic field measurements for any environment without the additional modifications because the software solution is designed so that the scale and corresponding color scale, linear or extremal opacity scaling, and the number of isosurfaces can be chosen for the magnetic field visualization. The limitation of the methodology is in the grid density because if a more dense measurement is performed, the more precise maps can be obtained; however, the time demands are significantly higher. Therefore, a convenient grid size has to be chosen according to the measurement purpose.

**Figure 13:**
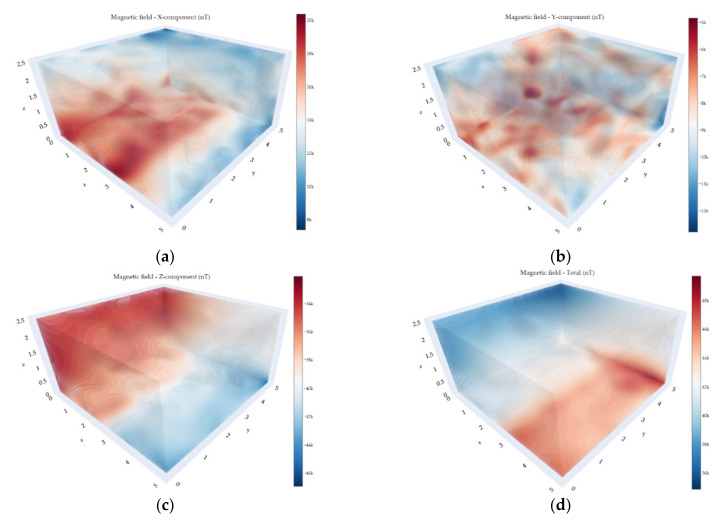
The DC (Direct Current) magnetic field measurements with the VEMA magnetometer: (**a**) X-component; (**b**) Y-component; (**c**) Z-component; (**d**) total.

One of the main advantages of using a fluxgate magnetometer, besides the precision of the measurements, is that the DC and AC magnetic fields are recorded together (DC—250 Hz bandwidth) at the same time. Figure 13 illustrates the spatial distribution of the DC magnetic field values obtained from the measurements. The measurements were performed in the afternoon after 3 p.m. Additionally, due to the current restrictions concerning COVID-19 involving work from home orders, the DC magnetic field could only be viewed as stationary. The zero value on the z-axis corresponds to the height of 0.5 m above the floor. The x, y, and z axes correspond to the distance from the origin of the coordinate system. The Y-component exhibited the most complicated spatial distribution among the components; however, in the entirety of the magnetic field, it is not very visible. All of the observed inhomogeneities in the DC magnetic field are caused by the local objects placed in the hall and by its construction.

**Figure 14:**
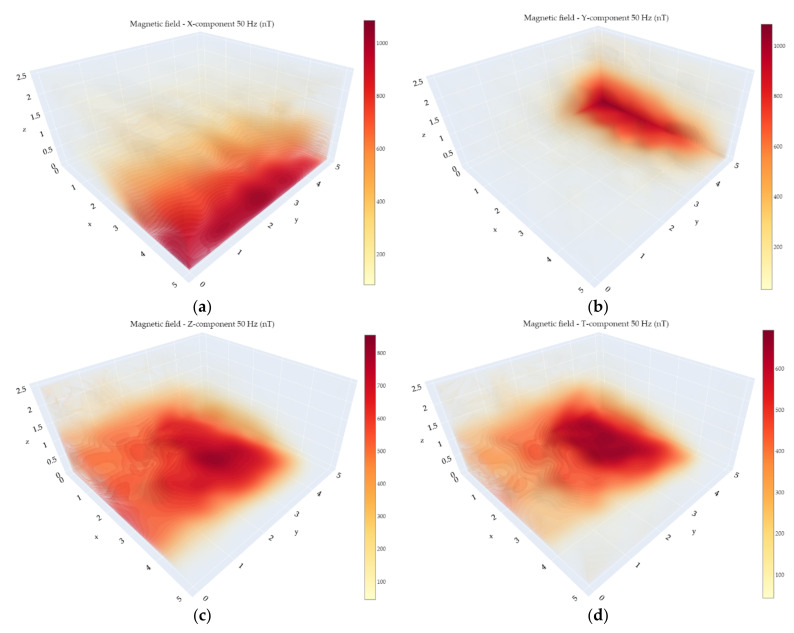
50 Hz amplitudes of the magnetic field measured with the VEMA magnetometer: (**a**) X-component; (**b**) Y-component; (**c**) Z-component; (**d**) total.

**Figure 15:**
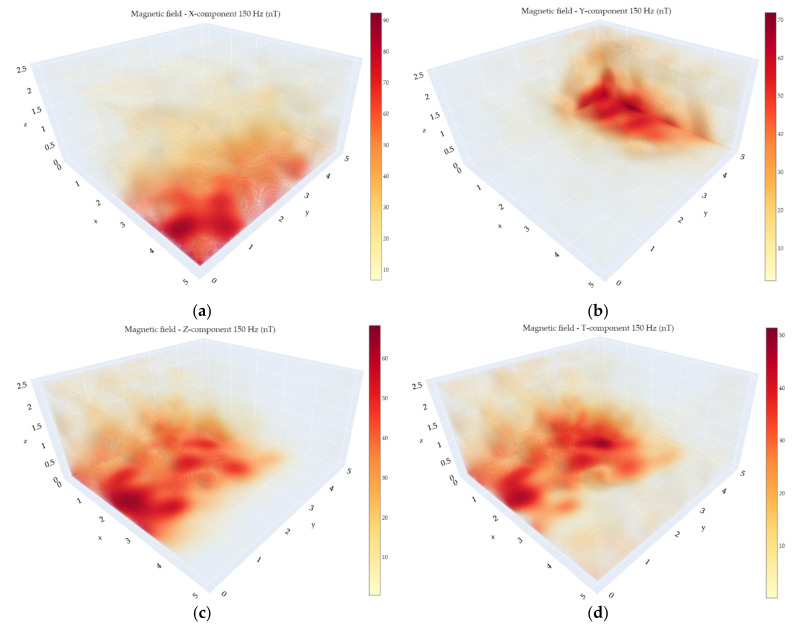
150 Hz amplitudes of the magnetic field measured with the VEMA magnetometer: (**a**) X-component; (**b**) Y-component; (**c**) Z-component; (**d**) total.

Since the bandwidth of the experimental VEMA magnetometer allows measurements of the 50 Hz industrial frequency and its 150 Hz harmonic, visualizations of their amplitudes were also created from the data records and can be seen in Figure 14 and Figure 15. The data recorded at every measurement point was processed by spectral analysis, and the industrial components for every point were determined. This is the reason why the position-hold mode for 2 s was used during the measurements performed using the UAV. In this way, it is possible to obtain much more precise information regarding the AC magnetic field in the measurement points. These calculated values were consequently interpolated and visualized applying the same methodology as in case of the visualization of the DC magnetic fields.

These frequencies were chosen since they are dominant in the background AC magnetic field. The lights were not switched on, so the part of the AC background was created by the cabling and pipeline channels under the floor leading to the neighboring building. The other part was created by the influence of this industrial magnetic field with the construction of the hall (mainly made from steel) and smaller objects and furniture placed behind the safety net. Additionally, as it can be seen in Figure 14, the 50 Hz component can be shifted in phase among the components, so the total field, if it was evaluated by a scalar magnetometer, does not reveal the fact that one component exceeds the amplitude presented in the total magnetic field.

An AC magnetic field with the frequency of 150 Hz can often tell us a lot about the “purity” of the power grid signal as well. Since there are reactance loads in the grid, and the grid phases are often not equally loaded, harmonics of the 50 Hz industrial frequency often occur. However, it is necessary to mention that these harmonics can also occur due to the spatial superposition of the fields, so the maximum of the 150 Hz signal does not always correlate with the maximum of the 50 Hz signal.

Considering the purpose of the indoor magnetic fields mapping, there are two main topics that have recently driven the research at the Department of Aviation Technical Studies. The first of which is navigation using the magnetic fields, and the second of which is technical cleanliness from the point of view of low frequency magnetic fields.

Each location or room in a building has its own specific magnetic field. The rooms differ not only in their position, size, and equipment but also in their magnetic image. The main background source of the stationary magnetic field is the Earth’s magnetic field and its variations, which can be regular and irregular. This background is often influenced and modified with the paramagnetic and ferromagnetic materials mainly used in construction. However, it is necessary to mention that the first modification is performed by the geological subsoil of the building. Other sources influencing the magnetic distribution are often furniture, other passive equipment, and active equipment in the room in the form of the power grid geometry and its loading with electrical appliances. This combination of sources often creates a unique magnetic image of the area of interest.

Navigation using magnetic fields can be viewed as correlation-extremal navigation. If the first position of the vehicle is known, we can correlate the trajectory of the known positions from the values of the measured DC magnetic field and estimate the current position. However, these algorithms are often demanding of the computational power that is available on the small UAV boards. Considering the possibility of measuring the low frequency magnetic fields up to the 250 Hz (as the experimental VEMA magnetometer), the industrial frequencies can also be used as a source of navigation information. This enables other dimensions for correlation algorithms if the magnetic map contains the spatial distribution of the mentioned AC components. However, since these AC component sources can depend on the power consumption of the devices loading the power grid of the buildings, it is necessary to choose sources (and frequencies) that can be considered as stationary. From this point of view, it seems to be more effective to evaluate the AC magnetic fields in a proportional ratio manner that includes normalization.

The technical cleanliness of workplaces/areas is gaining an increasing interest over the years, including from the electromagnetic point of view. The existence of an employee, a human, in these fields demands knowledge of these fields in space and time, creating an exposition image. The limit values are often specified in standards, guidelines, and recommendations issued by the appropriate bodies. However, the recommendations on which the standards are based differ in values, ranges, and the professional areas.

From this point of view, the presented visualizations provide a quick technical overview of the magnetic fields in the area since compared to the classic 2D slices, it is easier to resolve the information regarding the spatial distribution. Thanks to the variable opacity scaling of the isosurfaces, the depth perception of the volume is preserved, and the rendered graph can be freely rotated and zoomed, and thus speeding up the evaluation process for environmental purposes.

Considering the technical cleanliness or environmental issues that are gaining significant interest these days, for example, the ICNIRP (International Commission on Non-ionizing Radiation Protection) guidelines for low frequency magnetic fields [68,69,70,71,72,73] state the reference values as they are mentioned in Table 1 and Table 2, where the values are overviewed in the form of the unperturbed RMS (Root Mean Square) values. If we look at the table, for the industrial frequencies, the 1/*f* factor is omitted without stating a reason. 

One can argue that the 50/60 Hz magnetic fields have been in our environment for a long time, and humanity has adapted to them. This is probably true, but we have to consider the modern devices, often involving switching power sources, that can create harmonics of 50 Hz and even higher frequencies up to hundreds of kHz. If the 1/*f* factor was included, and the order increased to the 10^−3^ (as in the previous range), the limiting value for 50 Hz would be 40 µT RMS, which implies 56 µT amplitude. However, we have to consider the vector nature of the magnetic field and evaluate its components overtime and according to Equation (4), the superposition of the fields over wider frequency range:

(4) $$\sum_{j=1\;\mathrm{Hz}}^{10\;\mathrm{MHz}}\frac{H_{j}}{H_{R,j}}\leq 1,$$

where *H_j_* is the magnetic field strength at the *j* frequency, and *H_R,j_* is the magnetic field strength reference level at the *j* frequency as given in Table 1 and Table 2.

The limit values provided by the ICNIRP are rather high compared to the values stated, for example, by the recommendation of the EROPAEM guideline for the prevention, diagnosis, and treatment of EMF-related health problems and illnesses [74], summarized in Table 3. These values are recommended for the places where people spend more than 4 h per day (the frequency range is considered to be from 50 Hz up to 2 kHz in the source [74]).

From this point of view, indoor mapping using small UAV equipped with a fluxgate magnetometer with the sufficient range can measure the DC and AC components of the magnetic field in one measurement and can significantly help with the evaluation of the environment from the magnetic field perspective, especially in larger indoor areas where the GPS cannot be used. The ultrasound positioning system can be made portable on extendable rods and thus creates a relatively cheap positioning system for the UAV.

The contributions of the presented work can be found in several areas. In the hardware area, the paper presents a unique, however not complicated, complex magnetometric mapping system for indoor mapping using an ultrasound positioning system for the multirotor UAV that can be realized without high financial costs. In comparison to other published papers, the originality of the solution is that UAV can be used not only in the external environments, but thanks to the integration of the ultrasound positioning system, can be used for indoor magnetic field mapping, which can be relatively quick and processed either for the chosen altitude—this approach is usually also used in other papers dealing with the enhanced algorithms and systems for the UAV indoor navigation—or at multiple altitudes. In this way, it is possible to obtain a quick overview of the spatial distribution of the magnetic field, which can be very useful information not only for navigation purposes, for example, but also from the technical cleanliness point of view.

In the sensorics area, the system is compared to other studies capable not only of mapping the DC magnetic fields but also the AC magnetic fields up to the 250 Hz thanks to the simultaneous measurements of the AC and DC magnetic field components performed by the VEMA magnetometer.

The next contribution can be found in 3D visualization using isosurfaces, which gives an original technical insight into the spatial distribution of a magnetic field. The use of isosurfaces that are created by the connection of points with equal values preserves the depth perception in the volume and is easily understandable for technical personnel. This kind of visualization is also more suitable for the interpretation of the obtained data than volumetric rendering, which can introduce “foggy” boundaries and requires more computation power.

Forasmuch as the measurements were performed using the experimental version of the VEMA magnetometer, our future research will be focused on its optimization and miniaturization because with smaller dimensions and lower power consumption, it will be possible to use the payload more effectively and to achieve longer flight times. Considering the recently announced updates in autopilot software, future work will focus on implementing real-time magnetometer data correction based on the pitch, roll, and yaw angles obtained from autopilot as in-flight information thus providing significantly shortened time required for the mapping. Since there is ongoing parallel research at the department that is focused on the development of the magnetometers based on the magnetic microwires, for the future work, after finishing the development of the system, we intend to also use these systems on the UAV board. Continuous research will also be performed in the area of UAV customization, enhancement to increase the precision, and accuracy of magnetic field mapping for different application purposes.

## 4. Conclusions

In the civil sector, there are only a few studies dealing with the possibilities of indoor UAV use and are mainly focused on search and rescue applications, many times in unknown environments; therefore, the research in this area is focused on sense and avoid systems. In our case, the UAV was originally used for the indoor mapping of not only the DC but also for the industrial AC magnetic fields. For this purpose, the customized version of the quadcopter type UAV was designed and manufactured using a 3D printer. For indoor navigation purposes, an ultrasonic positioning system was integrated. In comparison to other available indoor navigation systems, it provides the cost-effective solution with sufficient precision.

In the role of the magnetic sensors, the originally developed experimental version of the fluxgate VEMA magnetometer, modified especially for this purpose, was used. The VEMA magnetometers allowed us to perform magnetic measurements with the sensitivity of 3.5 nT/LSB in the frequency range up to the 250 Hz, which makes it suitable for mapping purposes, even from the technical cleanliness point of view. Moreover, the VEMA magnetometer provides precise simultaneous measurements in all measurement channels, which could help to reveal non-stationary and time-varying magnetic fields that cannot be identified by scalar magnetometer or vector magnetometers with multiplexed channels. 

The obtained DC magnetic field measurement results were compared to the commercial LEMI-011 fluxgate magnetometer. The obtained results from the verification measurements proved the convenient precision, accuracy, and reliability of the VEMA magnetometer applicability on the UAV board. As with other magnetic field sensors used with UAVs, the VEMA sensors also need to be placed at the sufficient distance under the UAV to suppress the influence of the UAV’s construction; however, the necessary distance also varies with the power and efficiency of the electric propulsion system and the sensitivity of the measurement device. Due to the complexity of the UAV interference sources, it is usually necessary to determine the distance experimentally as in our case.

After the postprocessing of the obtained data, it is possible to create not only a 2D image of the magnetic field, but also a3D image of the magnetic field, offering quick technical insight into the spatial distribution of the magnetic field. The 3D graphs can be freely rotated and zoomed. This contributes to the increased readability and understandability of the created magnetic maps for the evaluating technical personnel. The results obtained from the spatial measurements can be further used for the correlation-extremal navigation using the different physical fields or for the evaluation of the magnetic fields in regard to the environmental engineering or technical cleanliness with respect to the human health.

As it can be seen from the achieved results, the measurements can reveal and identify sources of the magnetic fields, even if they are hidden, for example, by the construction of a building. The local anomalies of the magnetic field caused by the building construction can also be identified and suppressed or eliminated during reconstructions if necessary. Since vector magnetometers provide complete information about the decomposition of the local magnetic field vector into the orthogonal components, in comparison to scalar magnetometers, it is possible to determine magnitude, direction, and variation of the magnetic field, although they are not involved in the currently valid guidelines and directives.

## Figures and Tables

**Table 1:** Reference levels for occupational exposure to time varying electric and magnetic fields. Reprinted from ref. [70].

Frequency Range	*E*-Field Strength *E* (kV·m^−1^)	Magnetic Field Strength *H* (A·m^−1^)	Magnetic Flux Density *B* (T)
1 Hz–8 Hz	20	1.63 × 10^5^/*f*^2^	0.2/*f*^2^
8 Hz–25 Hz	20	2 × 10^4^/*f*	2.5 × 10^−2^/*f*
25 Hz–300 Hz	5 × 10^2^/*f*	8 × 10^2^	1 × 10^−3^
300 Hz–3 kHz	5 × 10^2^/*f*	2.4 × 10^5^/*f*	0.3/*f*
3 kHz–10 MHz	1.7 × 10^−1^	80	1 × 10^−4^

**Table 2:** Reference levels for general public exposure to time varying electric and magnetic fields. Reprinted from ref. [70].

Frequency Range	*E*-Field Strength *E* (kV·m^−1^)	Magnetic Field Strength *H* (A·m^−1^)	Magnetic Flux Density *B* (T)
1 Hz–8 Hz	5	3.2 × 10^4^/*f*^2^	4 × 10^−2^/*f*^2^
8 Hz–25 Hz	5	4 × 10^3^/*f*	5 × 10^−3^/*f*
25 Hz–50 Hz	5	1.6 × 10^2^	2 × 10^−4^
50 Hz–400 Hz	2.5 × 10^2^/*f*	1.6 × 10^2^	2 × 10^−4^
400 Hz–3 kHz	2.5 × 10^2^/*f*	6.4 × 10^4^/*f*	8 × 10^−2^/*f*
3 kHz–10 MHz	8.3 × 10^−2^	21	2.7 × 10^−5^

**Table 3:** Precautionary guidance values for the ELF (Extremely Low Frequency) magnetic fields. Reprinted from ref. [74].

ELF Magnetic Field	Daytime Exposure	Nighttime Exposure	Sensitive Populations
Arithmetic	100 nT	100 nT	30 nT
mean (AVG)	(1 mG)	(1 mG)	(0.3 mG)
Maximum	1000 nT	1000 nT	300 nT
(MAX)	(10 mG)	(10 mG)	(3 mG)
